# PReS-FINAL-2343: Cartilage thickness of the knee in juvenile idiopathic arthritis. comparative assessment by ultrasonography and magnetic resonance imaging

**DOI:** 10.1186/1546-0096-11-S2-P333

**Published:** 2013-12-05

**Authors:** D Pradsgaard, B Fiirgaard, AH Spannow, CW Heuck, T Herlin

**Affiliations:** 1 Pediatrics, MRI centre, Aarhus University Hospital Skejby, Aarhus, Denmark; 2 Diagnostic Radiology, MRI centre, Aarhus University Hospital Skejby, Aarhus, Denmark

## Introduction

The functional disability experienced in JIA is primarily caused by the degeneration of the osteocartilaginous structures due to the inflammatory process in the synovium. The ability to visualize the inflammatory process and the following osteocartilaginous degeneration is therefore of great importance in pediatric rheumatology. Magnetic resonance imaging (MRI) may currently be regarded as the Gold standard due to the ability to visualize all tissues with excellent precision. Ultrasonography (US) has been validated as a tool for measuring cartilage thickness in healthy children.

## Objectives

To validate and compare US with MRI measurements of distal femoral cartilage thickness in the knee joint. Further, to compare outcome measures of inflammatory joint activity, and bone damages in the knees of children diagnosed with oligoarticular JIA.

## Methods

Twenty-three children, median age 11.9 yrs (7.2-15.7 yrs), 17 girls and 6 boys with oligoarticular JIA where included. The knee joints were investigated by MRI and US. Outcome measures of clinical examination were distal femoral cartilage thickness, in addition to inflammatory outcome measures of joint activity, such as synovitis, effusion, bone marrow edema (MRI), and Color Doppler signal (US). A clinical examination registered objective signs of joint inflammation, swelling within the joint or limitation in the range of movement with pain or tenderness.

## Results

We found a high level of agreement between MRI and US measurements of mean cartilage thickness, when US measures were corrected for sound velocity in cartilage, and Rho values between modalities were high (between 0.70 and 0.86, p < 0.05 for all). MRI and US were superior to clinical examination in detection of joint inflammation. Level of agreement for detection of synovitis was high, however MRI was superior in detection of effusion.

## Conclusion

US measurements of distal femoral cartilage thickness is highly correlated to MRI measurements. MRI and US are superior to clinical examination in detection of inflammatory joint activity.

## Disclosure of interest

None declared.

